# The impact of China’s healthcare reforms on promoting early treatment for femoral neck fractures

**DOI:** 10.1097/JS9.0000000000001574

**Published:** 2024-05-09

**Authors:** Wenyi Gan, Tengfeng Zhuang, Chongxuan Lu, Minying Xiong, Songwei Huan

**Affiliations:** a Department of Bone and Joint Surgery, The First Affiliated Hospital of Jinan University, Jinan University; b The Second School of Clinical Medicine, Southern Medical University, Guangzhou, Guangdong, People’s Republic of China

*Dear Editor,*

Femoral neck fractures are highly prevalent among the elderly population and are usually closed fractures caused by falls or injuries requiring emergency medical attention. Due to differences in medical resources, treatment options, and healthcare processes across countries and regions, the time interval from patient admission to surgical treatment varies. However, an increasing number of studies have shown that early surgical intervention can effectively reduce the incidence of postoperative short-term complications in patients with femoral neck fractures^1^. Recently, I read an article published by Wang *et al*.^2^. This study, for the first time, utilized a national inpatient database in China and found that the proportion of Chinese patients with femoral neck fractures receiving early (within 48 h) hip arthroplasty slightly increased between 2013 and 2019. Compared to delayed surgery, both early total hip arthroplasty and early hemiarthroplasty were associated with lower risks of deep vein thrombosis and blood transfusion. Furthermore, early total hip arthroplasty was also associated with a lower risk of pulmonary embolism and a lower 30-day readmission rate. The study suggests that although the proportion remains low, early hip arthroplasty in China can significantly improve the prognosis of patients with femoral neck fractures, and more efforts should be made to optimize this surgical approach and shorten the waiting time for surgery.

Firstly, a retrospective analysis^3^ of 2041 high-cost hospitalization cases in 2016 revealed that factors such as insufficient use of high-value consumables, prolonged hospital stays, and unnecessary treatments drove up patient costs. In response, China’s healthcare reform has implemented a series of effective policies, including performance assessments of public hospitals based on hospital days and average costs per stay, promoting clinical pathways and single-disease management to standardize diagnosis and treatment processes, and improving the triage mechanism in emergency departments to reduce patient waiting times. Clinical pathways^4^ and single-disease quality management can reduce clinical variations, ensure medical quality, and improve diagnostic and treatment efficiency by standardizing diagnostic and treatment behaviors and refining treatment plans. The diagnosis and treatment of femoral neck fractures involve multiple aspects, including imaging, anesthesia, surgery, nursing, and rehabilitation, requiring close cooperation among relevant departments. Clinical pathways embed best practices into the healthcare workflow, clearly defining the tasks, standards, and timeframes for each step, strengthening multidisciplinary collaboration, minimizing decision-making and execution deviations, preventing delays and misdiagnoses, and ensuring that patients receive standardized diagnosis and treatment in a timely manner. Furthermore, China has vigorously implemented the Diagnosis-Related Group (DRG)^5^ payment policy and centralized procurement of drugs and consumables^6^, making efforts from both the medical insurance payment and supply guarantee ends to further curb the rapid growth of medical expenses and reduce the economic burden on patients, thereby increasing the willingness and accessibility of fracture patients to seek medical care. The DRG payment reform sets a fixed payment standard for each disease diagnosis, linking medical insurance payments with clinical pathways, forcing hospitals to control costs, standardize diagnosis and treatment, reduce the economic burden on patients, and improve patients’ accessibility to early medical treatment. These reforms have already shown initial results. A survey conducted in Wenzhou found that the implementation of these policies can motivate public comprehensive hospitals to improve their overall capabilities and narrow the gap in the efficiency of treatment costs for similar diseases^5^.

Secondly, enhanced recovery after surgery (ERAS) is a comprehensive perioperative management model that aims to reduce surgical trauma and complications, accelerate patient recovery, shorten hospital stays, and improve medical quality and efficiency by optimizing various aspects before, during, and after surgery. In recent years, the orthopedic community in China has also been actively promoting the ERAS concept^7^. An increasing number of tertiary hospitals have established ERAS centers and developed specialized ERAS programs for hip and knee replacements, achieving good results^7^. For patients with femoral neck fractures, ERAS helps optimize preoperative assessment and preparation, accelerate disease stabilization, perform surgery as early as possible, reduce bed rest time, and lower the risk of complications such as pressure ulcers, pulmonary infections, and lower limb deep vein thrombosis, thus improving the accessibility and safety of early surgery^7^. Day surgery refers to an advanced surgical management model in which patients are admitted, undergo surgery, and are discharged within 24 h. Currently, total hip and total knee replacements have become common types of day surgery performed internationally. Although patients with femoral neck fractures are mostly elderly, with multiple preoperative comorbidities and a high risk of postoperative complications, and have not yet been included in the scope of day surgery in many regions, it is believed that with the advancement of minimally invasive techniques, improvement of anesthesia levels, and refinement of rehabilitation measures, more and more low- to moderate-risk patients will be included in day surgery management through standardized screening in the future, further improving surgical and rehabilitation efficiency. Therefore, ERAS and day surgery, as important measures to improve the efficiency of medical services and enhance patient experience, are of great significance for further promoting early surgery for femoral neck fractures. This plays a positive role in further narrowing the gap between China and developed countries in this field.

Overall, although the implementation of early surgery for patients with femoral neck fractures in China is not as prevalent as in developed countries due to various constraints, with the deepening of healthcare reform and the overall improvement of medical service efficiency, China is gradually creating conditions for the standardized implementation of early surgery for fractures. In the future, with further optimization of medical resource allocation, deepening of medical insurance and medical service price reforms, and improvement of clinical practice guidelines, Chinese medical institutions will undoubtedly be able to better practice the concept of ‘early surgery,’ allowing the majority of patients with hip fractures to receive standardized diagnosis and treatment in a timely manner, improve their prognosis, and further narrow the gap in diagnosis and treatment compared to patients in developed countries.

## Ethical approval

Not applicable.

## Consent

Not applicable.

## Sources of funding

Not applicable.

## Author contribution

Guangzhou Science and Technology Plan Project, 2024A03J0757.

## Conflicts of interest disclosure

There are no conflicts of interest.

## Research registration unique identifying number (UIN)

Not applicable.

## Guarantor

Songwei Huan, e-mail: huansongwei123@163.com.

## Data availability statement

The data underlying this article will be shared by the corresponding author on reasonable request.

## Provenance and peer review

Not applicable.
